# Implementing ICT in classroom practice: what else matters besides the ICT infrastructure?

**DOI:** 10.1186/s40536-022-00144-6

**Published:** 2023-01-13

**Authors:** Catalina Lomos, J. W. (Hans) Luyten, Sabine Tieck

**Affiliations:** 1 Luxembourg Institute of Socio-Economic Research (LISER), Maison des Sciences Humaines, 11, Porte des Sciences, 4366 Esch-sur-Alzette, Belval, Luxembourg; 2 University of Twente, Enschede, The Netherlands; 3 Sampling Unit, International Association for the Evaluation of Educational Achievement (IEA), Hamburg, Germany

**Keywords:** ICILS 2018, ICT implementation model, Technology-driven approach, Pedagogical use of ICT, ICT resources, Teacher ICT self-efficacy, Nonresponse adjustment

## Abstract

**Background:**

The large-scale International Computer and Information Literacy Study (2018) has an interesting finding concerning Luxembourg teachers. Luxembourg has one of the highest reported level of technology-related resources for teaching and learning, but a relatively lower reported use of ICT in classroom practice.

**Methods:**

ICT innovation requires a high initial level of financial investment in technology, and Luxembourg has achieved this since 2015. Once the necessary financial investment in ICT technology has been made, the key question is what else matters to increase the use of ICT in teaching. To identify the relevant factors, we used the “Four in Balance” model, aimed explicitly at monitoring the implementation of ICT in schools.

**Results:**

Using data for 420 teachers in Luxembourg, we identify that within such a technology-driven approach to digitalization, teachers’ *vision of ICT use in teaching, level of expertise*, and the use of *digital learning materials in class* are significant support factors. *Leadership and collaboration*, in the form of an explicit vision of setting ICT as a priority for teaching in the school, also prove to be important.

**Conclusions:**

Through these findings, we show that the initial investment in school infrastructure for ICT needs to be associated in its implementation with teachers’ ICT-related beliefs, attitudes, and ICT expertise.

**Supplementary Information:**

The online version contains supplementary material available at 10.1186/s40536-022-00144-6.

## Introduction

The importance of ICT and teachers’ key role in integrating ICT in classroom practice has become even clearer since the COVID-19 crisis. The International Computer and Information Literacy Study (ICILS) performed in 2018 and covering 13 educational systems allows for an in-depth assessment of the relevant factors. Luxembourg, one of the countries participating for the first time in ICILS in 2018, provides an interesting finding. Based on the secondary schools and teachers included in the study, Luxembourg has one of the highest levels of technology-related resources available for teaching and learning, but a relatively lower reported teachers’ use of ICT in classroom practice. Only Denmark and Finland have a similar level for some of the ICT resources reported, but in both countries teachers report a high level of ICT use.

It is known that technological innovation has another important aspect that makes it more challenging than other educational innovations; that is, the necessary financial investment associated with it (Hassan & Geys, 2016). In ICILS 2018, Luxembourg is listed among the better resourced countries, in terms of the ratio of digital devices per student, the technology facilities available in secondary schools, and almost no computer resource hindrances to teaching and learning (Fraillon et al., 2020b). From 2014, Luxembourg began to guide its national policy toward digitalization outcomes, with the first (and very visible) measure being to equip schools with high-quality ICT resources and to provide all children with a computer or IPad (MENJE, 2015). Many digital learning programs, platforms, and online tools have since been developed and made freely available to teachers and students (MathemaTIC, 2015; MENJE, 2015).

Despite the high perceived availability and quality of ICT resources, ICT classroom practice is nevertheless at a low level, as identified in the ICILS study. Müller et. al. (2007) analyzed the socio-economic dimensions of ICT-driven educational change and concluded that the economic costs are the first step, but need to be followed by the social, human, professional, and institutional costs and involvement. The availability of Internet, hardware, and software are initially necessary, but need to be complemented by empowered schools and educational communities. More specifically, there is no longer a need to focus on the external (or first-order) barriers that could hinder the use of ICT, but attention could be shifted to the internal (or second-order) barriers. The second-order barriers are the skills, attitudes, and knowledge related to ICT classroom integration of those involved in this digital transformation (Hämäläinen et al., 2021; Makki et al., 2018). Many studies have investigated the factors related to teachers’ use of ICT in classroom practice (e.g., Afshari et al., 2009; Mumtaz, 2006; Spiteri & Chang Rundgren, 2020) and even in educational systems with an ample ICT infrastructure (Gil-Flores et al., 2017). First, Ertmer (1999) found that in addition to incremental or institutional characteristics (e.g., time, opportunities for professional development, and ICT as an institutional priority), fundamental or personal characteristics (e.g., vision, beliefs, and perceptions of utility or complexity) are also relevant supporting factors. Using the ICILS 2013 data, Gerick et. al. (2017) categorized the predictors of the use of ICT by teachers into: ICT equipment, professional development of teaching staff, school goals, teachers’ views on ICT outcomes, and their self-efficacy with ICT.

In the case of Luxembourg, where ICT was introduced as a top-down policy supported by initial strong investment, it is the implementation in schools that needs to be studied. In their meta-perspective paper on existing ICT strategies, Erstad et. al. (2015) identified one theoretical model that discusses the factors determining teachers’ use of ICT from an implementation perspective. This is the *Four in Balance* model (Kennisnet, 2011), which considers the factors for successful ICT implementation in schools, with the focus on the teachers’ use of ICT in classroom practice. The ICILS 2018 teacher data allows for the *Four in Balance* implementation model to be measured, using its latent scales as conceptual proxies, while investigating the following research question:

- What is the status quo of teachers’ use of ICT for classroom practice in Luxembourg?
- Which factors of the *Four in Balance* model are relevant for teachers’ use of ICT for classroom practice when ICT resources are not an issue?

The main purpose of this study is to identify important factors that account for variation in ICT implementation in a context of high availability of ICT resources. The findings of this study will indicate in what respect schools and teachers need support with regard to ICT implementation, when ICT resources are not an issue.

## The theoretical model

**Fig. 1:**
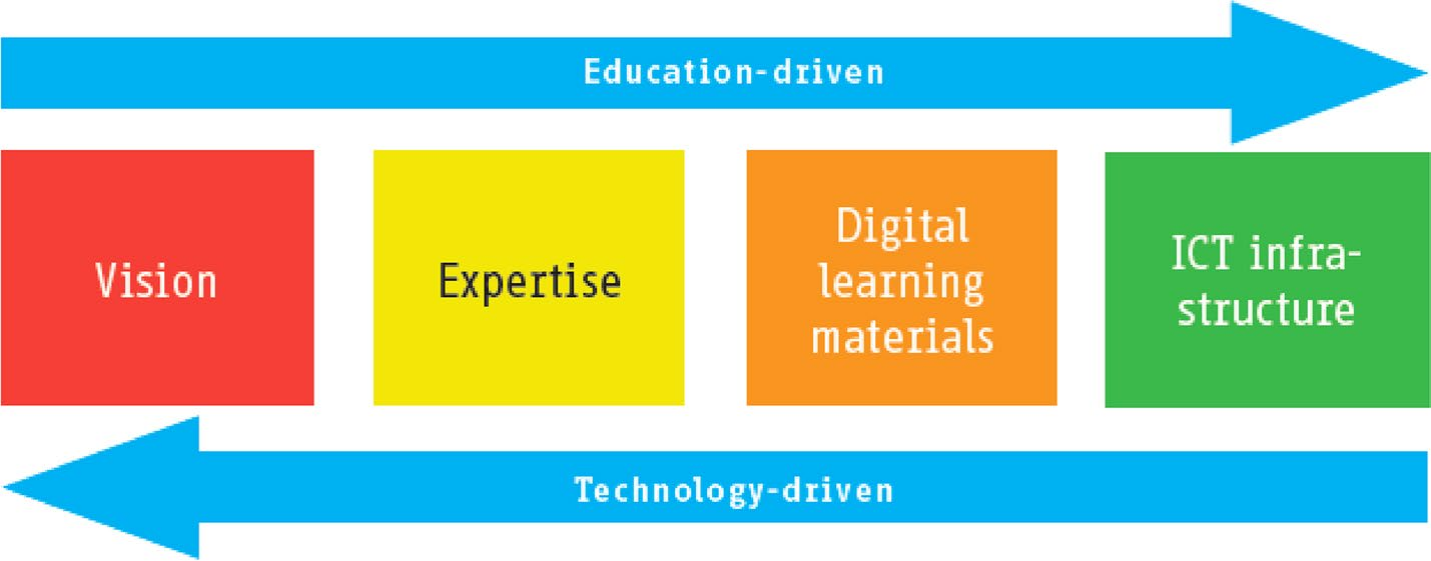
“Education-driven” versus “technology-driven” innovation in ICT (Source: Kennisnet (2011, p. 11, as Figure 1.2 in the source))

The *Four in Balance* model considers the factors for successful ICT implementation in education, approached through technology-driven or education-driven innovation (in Kennisnet, 2011; Koster et al., 2009; Law et al., 2008) (see Fig. 1). The underlying theoretical model was tested internationally and comparatively already in other countries, such as France, Germany, Japan, the Netherlands, Switzerland and the US (Tondeur et al., 2009; Tuijnman & Brummelhuis, 1992).

The technology-driven innovation in ICT starts with technology and digital learning materials, while the education-driven innovation starts with human factors, in terms of people’s vision and expertise. Considering these two approaches to ICT innovation, we conclude that Luxembourg embraced the technology-driven innovation approach in introducing ICT in schools, through its national strategy programs and the highly robust ICT infrastructure.

**Fig. 2:**
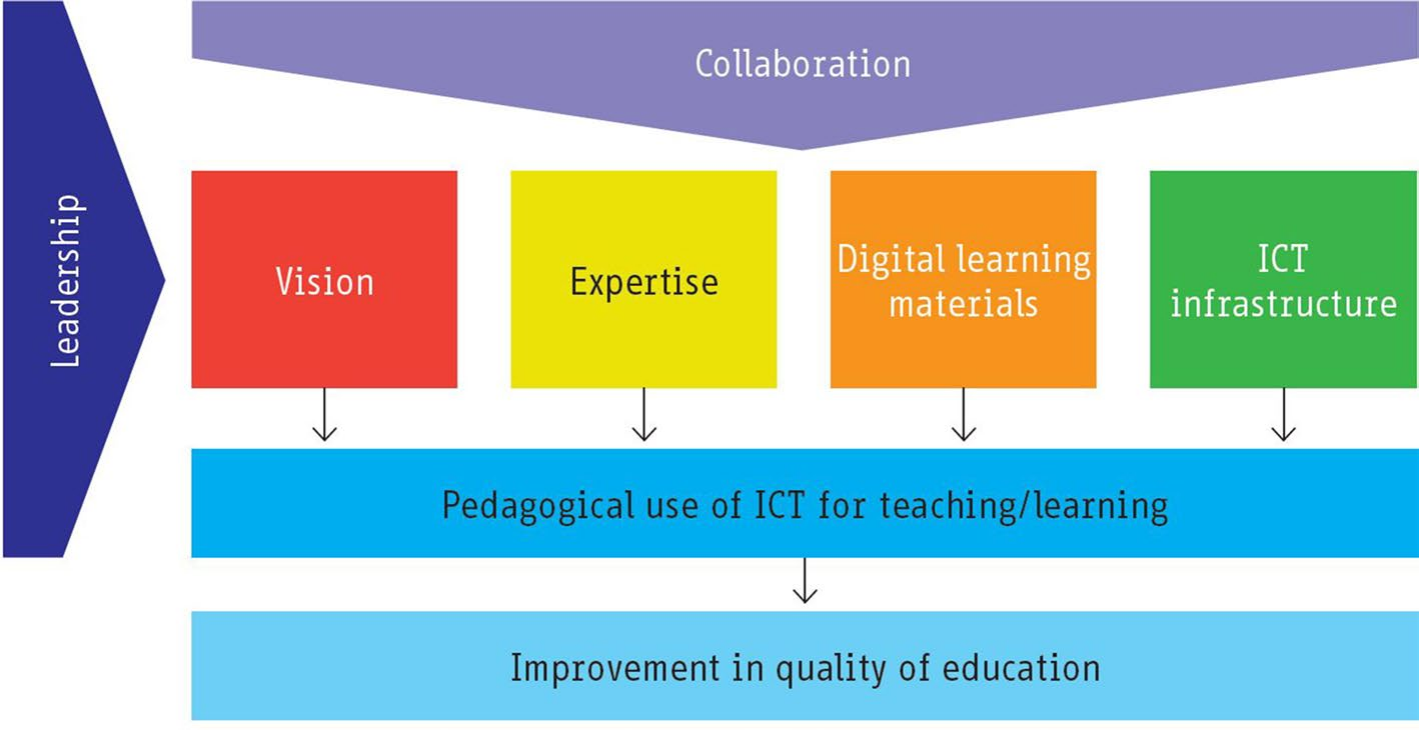
The basic elements of the Four in Balance model (Source: Kennisnet (2011, p.11, as Figure 1.1 in the source))

The *Four in Balance* model specifies four basic elements to support teachers’ pedagogical use of ICT, namely *ICT infrastructure, digital learning materials, vision*, and *expertise* (in Kennisnet, 2011; Brummelhuis, 2011) (see Fig. 2). *ICT infrastructure* refers to the availability of computers, access to the Internet, and all other similar facilities that relate with the use of ICT. *Digital learning materials* comprise all the digital educational content and tools that teachers’ use in their educational practice. *Expertise* relates to teachers’ knowledge and their technical and pedagogical skills in using ICT to achieve the educational objectives, while *Vision* refers to a school’s objectives and the role of teachers, students, and management in achieving specific ICT goals (Kennisnet, 2011, p. 9). Teachers play a crucial role in executing this model, in a close relationship with *leadership* and *collaboration* between professionals in the school.

Teachers’ perceptions of the *Four in Balance* model were identified and assessed in this study through the following aspects, presented here from the “technology-driven” innovation approach:

- ICT infrastructure—the availability of computers, interactive whiteboards, and Internet connection in their school.
- Digital learning materials—their use of computer programs in teaching and their use of digital learning materials from different sources;
- Expertise—their familiarity with ICT, level of skills for usage, and general pedagogical ICT skills;
- Vision—their pedagogical vision of using ICT for knowledge transfer and knowledge construction;

In addition, with regard to the general school context and facilitating conditions:

- Collaboration and leadership—teachers’ perceptions of collaboration between teachers, the support and source of support around ICT use, the presence of an ICT policy setting out the pedagogic vision for ICT, and the level of joint agreement regarding this ICT policy plan in the school.

*ICT infrastructure—*represented by computers, tools such as whiteboards, and a well-functioning Internet connection (Kennisnet, 2011)—is a necessary condition for teachers’ ICT use, but of course not a sufficient one in itself. The ICT infrastructure has proved to be the first and main barrier to teachers’ use of ICT for learning and e-learning, especially in many developing educational contexts (e.g., Auma & Achieng, 2020; Mailizar et al., 2020; Marwan, 2008; Owusu-Fordjour et al., 2020). Teachers need to have ready access to the necessary technology (Becker, 2000), and even more, they need to have the time and opportunities to use this infrastructure in their practice. Accordingly, we expect the availability of ICT infrastructure to support the use of ICT by teachers.

For *digital learning materials*, the *Four in Balance* model posits that the more teachers use digital learning tools and computer programs, the more they will tend to use ICT regularly for teaching and learning. Nevertheless, the use of digital materials and tools does not automatically imply the pedagogical use of ICT in the classrooms. The Technological Pedagogical Content Knowledge (TPACK) model defines the domains of expertise that are necessary for teachers’ pedagogical use of ICT. The model underlines the importance of mastering the technology through technological knowledge, technological content knowledge, and technological pedagogical knowledge, in order to integrate ICT pedagogically into classroom practice (Koehler & Mishra, 2005; Koh et al., 2013). Technological knowledge, represented by teachers’ skills in using digital tools and digital material, has proved to be a strong predictor for the use of ICT (Koh et al., 2013).

In terms of *expertise*, the *Four in Balance* model indicates that teachers’ familiarity with ICT, level of skills for its usage, and general pedagogical ICT skills are essential for its pedagogical use (Kennisnet, 2011). Teachers need to have personal experience of the technology, hands-on in teaching, for their intentions and actual behavior with ICT to increase (Ertmer, 2005; Kim et al., 2021). In fact, teachers’ initial professional development is seen as a precondition for them to learn how best to integrate ICT into their professional practices (Kopcha, 2012), to help them change their beliefs concerning ICT usage (Kim et al., 2012), and to make the effective steps for ICT implementation clearer (Thoma et al., 2017). In a meta-analysis study, Wilson et. al. (2020) conclude that pre-service training in ICT has been found to significantly increase teachers’ technical knowledge, even after only one course. Learning how to integrate ICT in teaching has also proved important, with the study by Wilson et. al. (2020) showing that combining theory and hands-on lab sessions has a significant effect in increasing technological knowledge. In line with this empirical evidence, we expect that, teachers who are more familiar with ICT and have higher levels of initial technological knowledge and ICT self-efficacy will use it to a greater extent in teaching and learning.

In terms of *vision*, the *Four in Balance* model indicates the importance of teachers’ beliefs and values regarding ICT, especially its role in knowledge construction. Knowledge transmission is the pedagogical approach through which teachers decide about and guide students in terms of when and how to learn, while through knowledge construction, teachers facilitate learning as part of an investigation process (Kennisnet, 2011). Schmid et. al. (2021) found that for planned and actual use of technology in education, teachers’ beliefs need to be considered and only a combination of such relevant predictors will be able to determine the differences in teachers’ use of technology. Kreijns et. al. (2013) found that teachers’ intention to use digital learning materials was determined by their attitude regarding its use and utility, and their general beliefs about teaching, among other things (Ertmer et al., 2012; Schimd et al., 2021). In line with this empirical evidence, our expectation is that teachers with more positive beliefs about ICT’s role in teaching are more likely to report high frequencies of pedagogical use of ICT.

*Collaboration and leadership*, as contextual characteristics, refer to teachers’ collaboration, their support through professional development within schools, and a joint agreement about ICT priorities in the school (Kennisnet, 2011). Empirical evidence has shown that relevant collaborative professional development can lead to improvement in teachers' use of ICT for instruction (Aldunate & Nussbaum, 2012; Kim et al., 2012), especially through collaborative forms of learning (Kopcha, 2012; Thoma et al., 2017). Teachers need to observe similar others (e.g., colleagues) carrying out the tasks and need access to multiple models in building their competence in order to change their intentions and behaviors. As Putnam and Borko (2000) note, teachers’ practice is more likely to change as they participate in learning communities that discuss new materials, methods, and strategies. In addition, a clear ICT policy plan and a centralized leadership ICT vision in a school (Eickelmann, 2011; Kennisnet, 2011) is necessary to accompany greater ICT use by teachers in their working practice. If schools lack a digital policy plan and a shared vision about why and how ICT needs to be integrated, they will miss the opportunity to support ICT teaching and learning in the school (Costa et al., 2021; Howard et al., 2020). Our expectation is that teachers who report a higher level of collaboration, a good level of support, and a clear centralized vision of ICT as priority in their school, will also report greater use of ICT in their working practice.

To summarize, our main hypothesis is that within a technology-driven approach to ICT innovation, teachers’ *expertise* and *vision* will vary more between teachers and will implicitly have a stronger significant relationship with the reported use of ICT in teaching.

## Context: ICT educational policy in Luxembourg

Luxembourg has a unique socio-economic context with direct implications for its educational system. First, Luxembourg’s small population is very diverse, as more than 45 percent of the population is of foreign origin. The largest foreign nationality group is the Portuguese, followed by the French and then other smaller groups of nationalities (Italian, Belgian, German, Balkan/Ex-Yugoslavian, British, other EU, and other non-EU). Moreover, Luxembourg is a trilingual country. Luxembourgish is the national language, French is used for legislation, and Luxembourgish, French, and German are used for social, administrative, and legal purposes (MENJE, 2020a). It should be noted that 43 percent of the pupils in public education do not have Luxembourg nationality and 62 percent of them do not speak Luxembourgish at home (MENJE, 2020a). These unique student demographics have an important impact on student performance in Luxembourg, as measured via large-scale studies in education, such as the PISA and the ICILS 2018 (Boualam, et al., 2021; SCRIPT, 2018).

In terms of ICT, Luxembourg ranks high as ninth on the ICT Development Index, with 97.8 percent of individuals aged 16–74 having used the Internet in the previous three months when surveyed (Fraillon et al., 2020b). Moreover, Luxembourg has placed a major focus on the ICT dimension at the political and social level. In 2014, Luxembourg launched *Digital Luxembourg*, a multidisciplinary government initiative to support digitalization for social and economic transformations (MENJE, 2015). This governmental action was transposed into education in 2015, with the launch of the *Digital (4) Education* national strategy. *Digital (4) Education* defined five major domains of competences: digital peer, digital citizen, digital entrepreneur, digital worker, and digital learner. These broad domains aimed to be achieved through various programs and projects. In ICILS 2018, however, with regard to the national curriculum for secondary schools, Luxembourg reported only an implicit emphasis on teaching aspects related to computer and information literacy and no emphasis on computational thinking (Fraillon et al., 2020b).

As the technology scene has evolved since 2018, Luxembourg has put in place a new strategy for digital education in schools. In 2020, a new digital approach termed *Einfach Digital* was introduced*,* being centered on five C’s: critical thinking, creativity, communication, collaboration, and coding (MENJE, 2020b). The core principles of this new approach for primary and secondary schools have been included in the *Guide de référence pour l’éducation aux/et par les médias* (SCRIPT, 2020). This framework and working document was the starting point for integrating media and digital competences in every day practice and guiding teachers in developing expertise and transferring new digital skills into teaching (Lomos, et al., 2021). Moreover, in 2021, a new subject was introduced in secondary education, termed *Digital Sciences* (MENJE, 2021) through a pilot phase with eighteen schools, which is intended to be embraced progressively by all schools and up to three grades by 2025.

The strategy *Einfach Digital*, introduced in 2020, endorses a skill-based perspective, transversal across subjects. It makes reference to digital citizenship and the production of information, with a focus on using the principles of technology and automation to develop skills to solve ecological, societal, and technological problems (MENJE, 2021). The wide availability of tailored professional development programs, conferences, practical sessions, presentations of pedagogical materials via exchange, and collaborative working groups across schools (MENJE, 2020b), offers space and support to teachers to integrate their attitudes, knowledge, and skills in this process.

## Method

A multilevel perspective on investigating the factors associated with the use of ICT in schools is facilitated by the assessment framework of the International Computer and Information Literacy Study (ICILS, 2018). ICILS 2018 offers a rich opportunity to identify and measure important ICT factors and their impact on ICT practice. Its numerous and comprehensive measurement scales make it possible to test relevant theoretical frameworks, being the *Four in Balance* theoretical model in this case.

### Variables

All the variables of interest in this study were addressed in the ICILS 2018 teacher questionnaire. All the data refers to teachers’ perceptions of the latent concepts analyzed.

Using the conceptualization of the four basic elements of the *Four in Balance* model, we selected the most appropriate scales from the ICILS 2018 teacher questionnaire, as the empirical assessment of the theoretical elements presented in Table 1. We prioritized the selection of scales over single items, when theoretically corresponding scales were available in the data. We selected three single items, not part of any scale, only when no scale was available to measure an aspect of the basic elements.

**Table 1:** The ICILS 2018 Teacher scales and single items selected as proxy measurements for the basic elements of the *Four in Balance* model

Four in Balance model—basic elements (definitions based on Tondeur et al., 2009, p. 392 & 393)	ICILS 2018 Teacher Scales (the items under these scales are presented in Appendix 1)
ICT infrastructure	
“[…] the right type of technology […]; sites where teachers and students can use them […]; using and maintaining ICT facilities […].”	ICT resources in the school (T_RESRC scale)
Digital learning materials	
"Educational software and content"; “[…] a spectrum of approaches to teaching and learning […]; teachers select applications of computers in line with their selection of other variables and processes […].”	Use of digital learning tools (T_USETOOL scale) Use of utility software (T_USEUTIL scale)
Expertise	
"Knowledge, attitude, and skills"; “[...] Professional development in the field of ICT means more than organizing training sessions for teachers to develop their technical skills. It is also about developing beliefs of teaching and learning and deliberately using ICT in the learning process.”	ICT use during lessons (single item) Initial teacher education on ICT (single item) Teachers’ ICT self-efficacy (T_ICTEFF scale)
Vision	
“[…] a shared vision of educational goals and structure is required. Such a vision consists of views about the roles played by teachers and pupils and the choice of methods and materials.”	Positive views on using ICT in teaching and learning (T_VWPOS scale)
Collaboration	
“Cooperation and support”; "[…] by sharing knowledge and materials, a common goal can be reached […]"; In turn, this contributes to the promotion of professionalism and a professional organization.”	Collaboration between teachers in using ICT (T_COLICT scale) Participation in collaborative PD learning (T_PROFREC scale)
Leadership	
“[…] The development of a vision on the use of ICT in educational practice means setting a direction for school development by identifying goals that are perceived as valuable by everyone involved in the ICT integration process.”	ICT as a school priority (single item)
Pedagogical use of ICT for learning	
“[…] good pedagogical use of ICT in the classroom.”	Teacher use of ICT for teaching practices in class (T_ICTPRAC scale)

The dependent variable is teachers’ pedagogical use of ICT, measured through eight items, each on a 4-point Likert scale. The independent variables we chose are in line with the *Four in Balance* theoretical model, capturing the four basic elements (ICT infrastructure, digital learning materials, expertise, and vision), guided through collaboration and leadership. Most concepts of interest were measured through scales of items, usually a set of 4-point Likert-scale items with responses ranging from low to high (strongly disagree to strongly agree; 1–4) (see Appendix 1). All items and scales were developed by the ICILS 2018 team based on a comprehensive assessment framework (Fraillon et al., 2019), built on previous theoretical developments and empirical evidence. Appendix 1 details the items and scales used in this study to test the theoretical model of interest, showing excellent proxy coverage of the theoretical model through these final selected ICILS scales. We considered the theoretical fit of other available scales as independent variables, but we did not include them in the analysis in the end. More specifically, the scales measuring teachers’ emphasis on ICT capabilities in class (T_ICTEMP) and of teaching coding tasks (T_CODEMP) were not included because of their explicit focus on very specific skills taught in class with the aim of supporting students to develop them.

Most of the items used in this study were integrated into Likert scales with high internal consistency (Cronbach’s alpha), as illustrated in Table 2. All the scales were built as IRT WLE scores with values on a continuum with an ICILS 2018 average of 50 and a standard deviation of 10, for equally weighted national samples (see the study’s Technical Report, Fraillon et al., 2020a). The scales were created by IEA (International Association for the Evaluation of Educational Achievement, in charge of the ICILS 2018 study) to allow for comparable results across countries in the study, and we accordingly chose to use the same scales to facilitate future applicability. We did not test the relationship of interest with individual items of these scales, and instead only focused on the relationships with the relevant validated scales.

**Table 2:** The scales used in the study to test the Four in Balance model

Scale	Number of items	Cronbach’s alpha	Mean	Standard deviation
Dependent variable				
Teachers’ use of ICT for teaching practices	8	0.83	45.54	9.38
ICT infrastructure				
ICT resources in the school	7	0.83	53.72	8.68
Digital learning materials				
Use of digital learning tools	10	0.78	44.41	8.58
Use of utility software	4	0.64	46.40	9.46
Expertise				
Teachers’ ICT self-efficacy	9	0.74	47.56	8.71
Vision				
Positive views about using ICT in teaching and learning	7	0.80	44.69	8.60
Collaboration and leadership				
Collaboration between teachers in using ICT	5	0.85	48.26	10.00
Participation in reciprocal PD learning related to ICT	4	0.72	47.90	9.41

*N* = 420 teachers; alternative weighting approach; the scales were built by ICILS as IRT WLE scores (Fraillon et al., 2020a) with M = 50, STD = 10

In order to appropriately test the *Four in Balance* model, relevant demographic characteristics were taken into account, in line with previous empirical evidence (Drossel et al., 2017; Siddiq & Scherer, 2016). The demographic characteristics tested are gender, age, and subject domain, with the expectation that female, younger, and mathematics and science teachers will report greater use of ICT for teaching and learning. Table 3 presents the frequency distribution for the categorical demographics and for the three single items selected to measure the domains of the model, in addition to the scales presented in Table 2. Dummy variables and their baseline categories are indicated, as are the single-item numerical variables, with their corresponding mean and standard deviation.

**Table 3:** Demographics and single-item variables used to test the Four in Balance model

Categorical Numerical	Percentage Mean (std. dev.)
Demographics	
Gender	
Female	58%
Male^a^	42%
Subject domain	
Math and sciences^a^	20%
Language and arts	45%
Human sciences	15%
Other	20%
Age (numerical)	41.10 (9.18)
Expertise	
ICT use during lessons	
Never	4%
Less than 2 years	14%
Between 2 and 5 years	22%
More than 5 years^a^	60%
Initial teacher training	
No ICT initial training^a^	63%
Learning how to use ICT or in teaching	15%
Learning how to use ICT and in teaching	22%
Collaboration and leadership	
ICT considered as a priority in teaching in the school (numerical) 1–4; strongly disagree–strongly agree	2.69 (.81)

*N* = 420 teachers; alternative weighting approach

^a^Baseline category for the regression analysis

### Sample

The analyses focus on teacher data from the ICILS 2018 study in Luxembourg, which was carried out as a census. All eighth-grade students from all secondary schools were selected to participate. Out of the 41 secondary schools in Luxembourg, 38 participated in the study, the other three declined to participate for different reasons. In each of the participating schools, a sample of teachers teaching eighth-grade students was selected. The number of teachers to select was increased to 25 instead of the requested minimum of 15, due to the small number of secondary schools in Luxembourg. In schools with fewer than 25 teachers, all of them were selected for participation. This resulted in 927 teachers from 38 schools selected to complete the teacher questionnaire. The participating teachers cannot be directly linked to individual participating students.

The ICILS 2018 followed a stratified^1^ two-stage sampling design, where first schools and then a certain number of teachers (within the sampled schools) were sampled. Two weighting factors are considered: the school base weight and the teacher base weight are the reciprocal of the respective sampling probability. A third weighting factor accounts for teachers working in more than one school.

Usually, not all schools or teachers are able or willing to participate, which results in nonresponse on the different levels. To account for this, two nonresponse factors—on the school and the teacher level respectively—were calculated. Non-participating teachers were taken into consideration by adjusting the weights of the participating teachers within the same schools (see Table 4). The nonresponse adjustment assumes that nonresponse is completely at random, which implies that within one school, participating and non-participating teachers are otherwise similar.

**Table 4:** Teacher nonresponse adjustment (WGTADJ2T) in international database ICILS 2018

Stratum No.	School No.	Nonresponse adjustment cell
Stratum 1	School 1	Participating teachers at school 1
Stratum 1	School 2	Participating teachers at school 2
Stratum 1	School 3	Participating teachers at school 3
Stratum 2	School 4	Participating teachers at school 4
Stratum 2	School 5	Participating teachers at school 5

Furthermore, in the international ICILS 2018 data, teachers in schools, where more than 50 percent of the teachers did not participate, were not included in the international database. Therefore, for Luxembourg 494 teachers from 28 schools were considered as participating in ICILS 2018. Moreover, ten participating schools had a participation rate lower than 50 percent. The implication is that if these teachers could be considered using an alternative weighting approach, we may obtain a better picture of the teacher population.

The final teacher weighting used in the international database is the product of all the teacher weighting factors. The calculation procedure for the final teacher weighting and for all the weight factors for the ICILS 2018 international database is described in the ICILS 2018 Technical Report (Fraillon et al., 2020a).

Although this is acceptable at the international level, we believe these teacher weights could be optimized for the Luxembourg national teacher data. Considering the high rate of nonresponse and the relevance of the sampling demographic characteristics to the outcomes of interest, teacher and school weights were re-estimated to work with a more precise population estimate for the teacher population. All the analyses and adjustments presented subsequently were performed with anonymized data for teachers and schools.

First, all 41 schools in Luxembourg were asked to participate, thus the teacher sample was a stratified systematic sample of all teachers. Stratified, because within schools, teachers were systematically sampled from a list sorted by gender, year of birth, and subject domain. This ensures a proportional teacher sample according to these characteristics, and allows us to define an alternative weighting approach, in which these characteristics are used to create alternative nonresponse cells.

Second, based on an analysis of the international data, we observe that teachers in the same age group, with the same subject domain, and within certain types of schools, give similar answers to the questionnaire. This is not the case for the dependent variable in the current study, but for other independent variables of interest (e.g., gender and collaboration between teachers using ICT, teachers’ use of digital tools in class, teachers ICT self-efficacy, age and experience with ICT use during lessons, positive views on using ICT in teaching and learning, use of ICT utility software, teacher participation in participative professional development, etc.). Thus, we believe that by defining the adjustment cells for non-participating teachers in line with these characteristics, the weights generated using this alternative weighting approach are better optimized for teacher analysis in Luxembourg.

Technical details of the re-weighting procedure and of the final teacher and school weights used in this alternative weighing approach are presented in Additional file 1.

### Data analysis

Multiple linear regression analysis is used to determine the relationship among the *Four in Balance* selected variables and teachers’ pedagogical use of ICT. The dependent variable *Teacher use of ICT for teaching practices in class (T_ICTPRAC)* had 64 cases (13%) with “missing by design” values (meaning “practice not applicable at this class”), their deletion resulting into a working sample of 430 teachers. After deleting other missing “98—not applicable” values in some of the independent variables used, we worked with a final sample of 420 teachers in 28 schools.

Multilevel data analysis was considered in line with the theoretical model, but the Intraclass Correlation Coefficient (ICC) showed that the bulk of the variance to be explained in teachers’ use of ICT is between teachers and not between schools (ICC = 0.05). Consequently, Multiple Linear Regression Analysis was used to test the *Four in Balance* model on our data for the 420 teachers in 28 schools in Luxembourg. SPSS software was used for data management, together with the IEA’s IDB Analyzer to perform the regression analysis, accounting for the specifics of the ICILS/IEA large-scale data. The IEA’s IDB Analyzer takes into account information from the sampling design in the computation of sampling variance, statistics, and standard errors. Moreover, it makes use of appropriate sampling weights. Standard errors for the statistics are computed according to the variance estimation procedure required by the design of the corresponding study (IEA IDB Analyzer, version 4.0). Our analysis in this way takes into account the nested structure of teachers within schools, and corrects the standard errors for the clustered sampling design (Fraillon et al., 2020a). Moreover, through weighting, the software also corrects for the more general sampling design in relation to the population of interest. However, in this study, we used the teacher and school re-estimated weights through the alternative weighing approach to try to account for teacher nonresponse, in order to produce more accurate estimates of the teacher population.

We tested different regression models separately, to identify the effect and the variance explained by the different factors/domains of the theoretical model. More specifically, we first tested a *demographics* model, by considering only the demographic characteristics. These demographic variables were kept as antecedents in all the following models tested. An *ICT infrastructure* model, a *digital learning materials* model, an *expertise* model, a *vision* model, and a *collaboration and leadership* model were tested separately in order to identify the contribution and effect of each domain on the dependent variable. Lastly, a *final model* was tested, taking into consideration all the significant variables identified previously. Standardized regression coefficients and their standard errors are reported, together with the explained variance R-square adjusted for each domain of the *Four in Balance* proxy model and the *final model*.

## Results

This section presents the results, in line with the *Four in Balance* theoretical model and its application to the ICILS 2018 data for Luxembourg.

Regarding the first research question on the status quo of teachers’ ICT use in practice, the dependent variable—the *pedagogical use of ICT*—scale has a mean of 45.54 (SD = 9.38) on a scale with a mean of 50 and standard deviation of 10, for equally weighted countries. When looking at the specific items of the scale, we can see that there is a relatively small percentage of teachers who report the use of ICT in often or always in their lessons for specific teaching practices (see Table 5).

**Table 5:** Pedagogical use of ICT

Pedagogical use of ICT	Percentage (%)
Percentages of teachers who reported the use of ICT (often or always) in their lessons for different teaching practices (excluding the teachers who did not use this practice with the reference class)	
The provision of remedial or enrichment support to individual students or small groups of students	45
The support of student-led whole-class discussions and presentations	36
The reinforcement of learning of skills through repetition of examples	29
The support of inquiry learning	25
The support of collaboration among students	16
The assessment of students’ learning through tests	15
The provision of feedback to students on their work	14
The mediation of communication between students and experts or external mentors	11

*N* = 420 teachers; alternative weighting approach; teacher practices ordered by the size of the percentage

Most of the practices used are in line with the aim of knowledge transmission: providing remedial or enrichment support, whole-class discussions and presentations, and reinforcement of learning through repetition of examples. Less frequent are practices using ICT to support collaboration between students or to provide feedback about their work.

To answer the second research question, the results of the regression analysis are presented in Table 6 with the details presented next, by model.

**Table 6:** Regression analysis coefficients

Effect Betta coefficient (S.E.)	Demographics	ICT infrastructure	Digital learning materials	Expertise	Vision	Collaboration and leadership	Final model
Intercept							
Demographics							
Gender (Male)	0.05 (0.06)	0.05 (0.06)	− 0.02 (0.04)	− 0.01 (0.05)	0.03 (0.04)	0.05 (0.05)	− 0.02 (0.04)
Age	− 0.07 (0.04)	− 0.07 (0.04)	− 0.05 (0.04)	0.02 (0.04)	**−** 0.10 (0.04)*	− 0.06 (0.03)	− 0.04 (0.04)
Subject (Math and sciences)							
Language and arts	− 0.04 (0.07)	− 0.03 (0.07)	− 0.04 (0.06)	0.02 (0.07)	− 0.09 (0.07)	0.00 (0.07)	− 0.03 (0.07)
Human sciences	− 0.03 (0.05)	− 0.04 (0.05)	0.00 (0.05)	0.02 (0.05)	− 0.05 (0.05)	− 0.01 (0.05)	0.01 (0.05)
Other	− 0.04 (0.06)	− 0.03 (0.06)	0.06 (0.05)	0.02 (0.06)	− 0.06 (0.05)	0.02 (0.06)	0.06 (0.05)
ICT infrastructure							
ICT resources in the school		0.15 (0.06)*					− 0.07 (0.05)
Digital learning materials							
Use of digital learning tools			0.38 (0.07)**				0.27 (0.07)**
Use of utility software			0.28 (0.06)**				0.17 (0.06)**
Expertise							
ICT use during lessons (more than 5 years)							
No earlier experience				**−** 0.16 (0.08)*			− 0.09 (0.07)
Less than 2 years				− 0.06 (0.06)			− 0.04 (0.04)
Between 2 and 5 years				− 0.06 (0.04)			− 0.05 (0.03)
Initial teacher training in ICT use (no ICT initial training):							
Learning how to use ICT or in teaching				0.06 (0.06)			0.02 (0.04)
Learning how to use ICT and in teaching				0.12 (0.05)*			0.03 (0.05)
Teachers’ ICT self-efficacy				0.35 (0.05)**			0.10 (0.05)*
Vision							
Positive views on using ICT in teaching and learning (knowledge construction)					0.38 (0.06)**		0.17 (0.05)**
Collaboration and leadership							
Collaboration between teachers in using ICT						0.18 (0.07)**	0.05 (0.06)
Participation in collaborative PD learning related to ICT						0.15 (0.05)**	0.06 (0.04)
ICT considered as a priority in teaching in the school						0.26 (0.06)**	0.13 (0.06)*
Model							
R-square adjusted	0.01	0.03	0.33	0.21	0.15	0.19	0.42
N	420	420	420	420	420	420	420

*N* = 420 teachers; alternative weighting approach; BETA standardized linear regression coefficients; p < 0.05* (two-tailed); p < 0.01** (two-tailed)

### Demographic characteristics

We see that gender, age, and the subject domain have no significant relationship with teachers’ reported use of ICT for educational purposes in their classes. Considering that these variables might produce spurious effects of other variables on the pedagogical use of ICT, we will keep these variables in all the models presented subsequently.

### ICT infrastructure

The *ICT infrastructure* model has a minor explanatory role (R-square = 0.03). We see a small but significant effect, with available ICT resources for teachers proving to be a significant positive predictor (β = 0.15, SE = 0.06). When investigated item by item for understanding purposes, time and opportunity for developing ICT expertise prove to be more important for teachers’ use of ICT in practice than the availability of computers and a good Internet connection.

### Digital learning materials

The use of *digital learning materials* during lessons proves to be a strong predictive model for teachers’ pedagogical use of ICT (R-square = 0.33). When teachers report more use of digital learning tools (β = 0.38, SE = 0.07) and of general utility software (β = 0.28, SE = 0.06), they also report greater use of ICT for their teaching and learning. Both the use of digital learning tools (*r* = 0.52, se = 0.06) and the use of general utility software (*r* = 0.46, se = 0.05) have a strong correlation with the dependent variable. However, the conceptual meanings are different. The dependent variable refers to pedagogical practices with ICT (e.g., remedial or enrichment support, student assessment, feedback, collaboration, and inquiry learning), whereas the other two scales simply list the digital tools (e.g., digital learning games and e-portfolios) and software (e.g., Word and wikis) teachers make use of in their work.

### Expertise

The model measuring *expertise* obtains an R-square of 0.21, the variable with the strongest effect being teachers’ ICT self-efficacy (β = 0.35, SE = 0.05). Moreover, initial teacher training in using ICT and using ICT in teaching has a significant positive effect (β = 0.12, SE = 0.05) compared with no such initial training. By comparison, never using ICT during lessons has a negative impact (β = − 0.16, SE = 0.08) when compared with using ICT during lessons for more than 5 years.

### Vision

We see that teachers’ *vision* about the role of ICT and its use in teaching and learning has an important explanatory value for teachers’ pedagogical use of ICT (R-square = 0.15). Teachers’ positive views about the outcomes of using ICT in education (β = 0.38, SE = 0.06), and especially about using ICT in knowledge construction practices (e.g., developing skills in planning and self-regulating work, developing problem-solving skills, and collaborating more effectively), are strongly and positively related with teachers’ reported pedagogical use of ICT.

### Collaboration and leadership

The model for *collaboration and leadership* obtains an R-square of 0.19, containing two scales measuring collaboration and one scale measuring leadership priority for ICT in the school. Greater reported collaboration between teachers using ICT (β = 0.18, SE = 0.07) and more reported participation in collaborative professional development learning related to ICT (β = 0.15, SE = 0.05) are both significantly associated with greater self-reported pedagogical use of ICT by teachers. In addition, when teachers perceive greater emphasis by the leadership in their school on ICT as a priority in teaching (β = 0.26, SE = 0.06), they also report more use of ICT in their classroom practice.

### ***Final model***

In the *final model*, we consider all the significant variables from the four previous models together, as presented in Table 6. We find an R-square of 0.42 and three domains appear significant: *digital learning materials, expertise*, and *vision*. In addition, the variable leadership agreement on ICT being considered a priority for teaching in the school also appears as significant in the final model.

In terms of the use of *digital learning materials*, the use of digital learning tools (β = 0.27, SE = 0.07) and of utility software in their classes (β = 0.17, SE = 0.06) prove to positively relate with teachers’ pedagogical use of ICT. With regard to *expertise*, teachers’ ICT self-efficacy (β = 0.10, SE = 0.05) has a significant positive relationship with the dependent variable. In terms of *vision*, teachers’ holding positive views about using ICT in teaching and learning (β = 0.17, SE = 0.05) is a significant predictor of the dependent variable. Lastly, one variable from the *leadership* domain—ICT being considered as a priority in the school (β = 0.13, SE = 0.06)—also proves relevant for teachers’ use of ICT in practice. None of the other variables, especially the collaboration domain variables, are significantly related anymore with the dependent variable. This suggests many other possible indirect paths between these variables that could be hypothesized and tested more thoroughly in future studies.

Table 7 presents a summary of the results, showing the R-squared adjusted of each single domain model and of the entire theoretical model.

**Table 7:** Explained variance (R-squared adjusted) of the models—nonresponse cross-validation

Four in Balance proxy regression models	Explained variance with the alternative final teacher weight	Explained variance with the international final teacher weight
ICT infrastructure	0.03	0.03
Digital learning materials	0.33	0.30
Expertise	0.21	0.18
Vision	0.15	0.15
Leadership and collaboration	0.19	0.16
Final model	0.42	0.38

*N* = 420 teachers

We can see again that together in the *final model*, the *Four in Balance* proxy model explains 0.42 of the variance of teachers’ pedagogical use of ICT, with *digital learning materials, expertise,* and *vision,* playing the most important roles.

The final teacher weight of the alternative weighting approach used here gave a similar population estimation to that of the final teacher weights in the international ICILS 2018 database. The variance explained by some of the models tested changed slightly. Table 7 shows the explained variance of the models (R-squared adjusted) obtained using both approaches. The change that deserves meaningful mention is the relatively smaller value of the explained variance for the *expertise* model when using the international database weights, model that tests for variables closely related with age (e.g., earlier experience with ICT use during lessons and teachers’ ICT self-efficacy scale).

## Discussion

The current study has looked at the factors identified in previous empirical investigations from the perspective of ICT implementation in schools within an educational system characterized by a high initial ICT technology support. We find that high financial investment in ICT for schools and communities becomes a support condition only when other staff-related and context-related factors are in place.

Again, the scale mean of the availability of ICT resources in schools in Luxembourg (M = 53.72, SD = 8.7) compared with that across all countries in ICILS 2018 (M = 50.0, SD = 10.0) shows a clear teacher agreement on the availability of ICT equipment, digital learning resources, and even the time and opportunity to prepare and develop expertise in ICT.

However, regarding the level of teachers’ pedagogical use of ICT, we find that many teachers in Luxembourg report using ICT in most lessons, but particularly for knowledge transmission (Kennisnet, 2011). More specifically, ICT is mostly used often or always in the provision of remedial or enrichment support to individual students or small groups (45 percent of teachers) and as a support in student-led whole-class discussions and presentations (36 percent of teachers), followed by the reinforcement of learning of skills through repetition of examples (29 percent of teachers). Fewer teachers in Luxembourg report using ICT in most lessons with the aim of knowledge construction: specifically, for inquiry learning (25 percent of teachers) or provision of feedback to students about their work (14 percent of teachers). The other teaching practices indicated, such as assessment of students’ learning through tests (15 percent of teachers) or the support of collaboration among students (16 percent of all teachers) can be supported through ICT, but not many teachers report this for most lessons, although it could be justified for some of these types of practices, such as tests.

These results indicate that teachers are still in an initial stage of ICT integration in teaching practices, making use of it for transmitting information and not yet for constructing more abstract knowledge. This indicates why it is very important to address the second research question and identify which less-tangible factors support teachers’ use of ICT in educational practice in Luxembourg.

First, it is important to mention that none of the demographic characteristics tested have a significant relationship with teachers’ use of ICT in pedagogical practice. Based on previous empirical evidence (Drossel et al., 2017), we would expect age and subject domain to have a significant relationship, but this is not found in our sample. There are other studies that have not found a significant association between age or gender, and teachers’ use of ICT or their ICT self-efficacy (Hämäläinen et al., 2021; Hatlevik & Hatlevik, 2018). A similar lack of association has been noted among pre-service teachers (Tondeur et al., 2018). In our findings, age becomes significantly related with teachers’ ICT use in practice only in the *vision* model, hinting at a possible interaction between age and teachers’ views on the utility of ICT. This is something that could be further investigated in future research.

In terms of *digital learning materials*, we expected that using simple software or digital learning platforms would be a precursor for the pedagogical use of ICT for remedial support, provision of feedback, inquiry learning, and more. The data analysis provides strong support for this expectation. The use of digital learning tools and of utility software, as an indicator of their technological knowledge, are strong predictors of teachers’ reported ICT use in practice (R-square = 0.33). What is surprising, however, is the relatively lower level of the reported use of digital learning tools, when comparing the scale mean in Luxembourg (M = 44.4, SD = 8.6) with the scale mean across all countries (M = 50.0, SD = 10.0). Luxembourg has developed and made available to teachers a large number of high-quality digital learning platforms and tools (MENJE, 2015, 2020b). This again proves to be a necessary but not a sufficient condition for actual use in practice. In turn, this implies that more effort is needed to support teachers in using these digital learning tools in practice (e.g., digital learning games, concept mapping software, simulation and modelling software, a learning management system, etc.), in order to increase the pedagogical use of ICT.

In terms of *expertise*, as expected, all the variables measuring different aspects of expertise show significant relationships with ICT use in practice: specifically, teachers’ use of ICT during lessons, initial teacher training with ICT, and ICT self-efficacy. First, the teachers with no earlier experience of ICT use during lessons, report the lowest pedagogical use in practice when compared with teachers having five or more years of experience. Teachers that never used ICT in teaching practice represent only 4 percent of the teachers in the present data, while most teachers (60 percent) report five or more years of experience. In terms of Initial training with ICT, only teachers who report having followed initial training for using ICT and using ICT in teaching (22 percent) report significantly more use of ICT in teaching when compared with those with no initial training (63 percent). Initial teacher training in ICT proves essential, as expected (Kopcha, 2012), but it is important to focus not only on ICT per se, but also on ICT in teaching. However, teachers’ perceived ICT self-efficacy shows the most important contribution in this model, being strongly and positively related with their use of ICT in educational practice (β = 0.35, SE = 0.05). Teachers who report a higher level of ICT self-efficacy also report a greater use of ICT in practice (Drossel & Eickelmann, 2017). This result confirms the expectation that teachers’ perceived ICT self-efficacy influences their intention to use ICT for teaching (Hatlevik, 2017; Teo, 2008), as well as their perceived use of it (Paraskeva et al., 2008).

In terms of *vision*, the findings are in line with our expectations. There is a lower level of agreement with the possible positive effects of using ICT in teaching in Luxembourg (M = 44.7, SD = 8.6) when compared with the set mean score of the scale across all participating countries (M = 50.0 SD = 10.0). Moreover, teachers who agree with the potentially positive outcomes of using ICT in practice also report greater use of ICT in teaching (β = 0.38, SE = 0.06). The scale of negative views on using ICT in teaching and learning is also part of the data, but it was not included in this model because of its high correlation with the scale of positive views. When introduced in the model, however, the scale of negative views also shows a significant negative association with teachers’ reported use of ICT in practice This association could also explain some reluctance in terms of conscious choices for the use (or non-use) of ICT and draws the attention toward the perception of the appropriate use of ICT.

In terms of *collaboration and leadership*, as reported by the teachers, we see that all variables are significantly related to teachers’ use of ICT in practice. Collaboration between teachers in using ICT proves to be a facilitator of its use, in line with previous research (Kopcha, 2012; Thoma et al., 2017). If ICT is considered a leadership priority for teaching in school, this also proves an important stimulator for ICT use in teaching practice. However, the mean of this scale indicates a low level of teachers’ agreement on ICT being considered a priority at their school, with only 58 percent of the participating teachers agreeing or strongly agreeing. This is an important aspect, considering that Ertmer (1999) and Drossel et. al. (2017) found that having a common school vision, especially one with ICT as a priority, is a predictor of teachers’ ICT use in practice in many countries. Liu et. al. (2020) also concluded their extensive review of 131 articles with a message “for institutional leaders on the importance of a clear expression of strategy intent supplemented by established decision making mechanisms and support” (p. 12).

The *final model* brings together all the significant predictors selected in the *Four in Balance* model and the different domains. We find that the *digital learning materials* domain, with its use of digital learning tools and utility software as a reflection of teachers’ technological knowledge, is the main predictor of teachers’ use of ICT in practice. It is followed by the *expertise* and *vision* domains, with an important contribution of teachers’ agreement with the positive outcomes of using ICT, and teachers’ ICT self-efficacy. Lastly, *ICT being considered as a priority in teaching in the school*, representing the *collaboration and leadership* contextual domain in the final model proves to also support teachers’ use of ICT. Following a 3-year longitudinal study, Müller et. al. (2007) recommended that teachers need space and time for exchange and debate, guided by clearly formulated goals and visions for the future. If we reflect back on the barriers that could hinder the use of ICT in teaching and learning, we see that our model identifies many second-order barriers as relevant, such as attitudes, self-perceived competence, and skills (Hämäläinen et al., 2021). These could be focused on in future strategies.

## Conclusion

Our findings enrich the knowledge of what factors need to be enhanced when implementing ICT in schools, within a technology-driven approach to innovation. Even though *ICT infrastructure* (MENJE, 2015) and *digital learning materials* have been made available to secondary schools in Luxembourg on a very wide basis, we see that teachers still differ in their level of use of these in practice. As in many TPACK studies (Howard et al., 2020), teachers’ technological knowledge—represented here by their use of digital tools and digital learning materials—proves to be a direct and important predictor of their use of ICT in classroom practice. Teachers’ *vision* concerning the positive outcomes of using ICT and integrating it in teaching, especially for knowledge construction, needs to be supported to a greater extent. Schmid et. al. (2021) also found that for the planned and actual use of technology in education, teachers’ vision and beliefs need to be considered, and only a combination of relevant predictors will be able to determine the differences in teachers’ use of technology. Teachers’ perceived ICT self-efficacy and long-term use of ICT in practice also need to be facilitated within schools.

To summarize, we find teachers’ *vision* and *expertise* concerning ICT to be key differentiators of its use in teaching; an important key message for all educational systems that take a technology-driven approach to innovation. At this stage in the process, more attention needs to be paid to understanding and guiding teachers’ vision regarding the use and utility of ICT in teaching practice, as well as teachers’ ICT self-efficacy and use. This could be achieved through a common school vision and through a common agreement that ICT is considered a priority in school. Policy directions, such as an explicit ICT-focused curriculum, need to be embraced and implemented in each school, through a common school vision on ICT, to actually transfer the impact into teachers’ vision and use of digital learning materials in class.

The high demand for ICT use in teaching during the COVID-19 health crisis has affected how teachers will use ICT in the future. The ICILS 2020 teacher panel (Strietholt et al., 2021) surveyed the same teachers as in the ICILS 2018 in three countries (Denmark, Finland, and Uruguay) and found a substantial increase in the use of ICT for learning, measured through reported frequency of use. Moreover, the study found little reported change in teachers’ vision measured through their positive and negative views on using ICT for teaching and learning. This result shows that the COVID-19 health crisis might have accelerated the accumulation of ICT skills, but did not necessarily contribute to improving teachers’ vision regarding the use and utility of ICT in practice.

## Limitations

These results offer opportunities for further research, especially for clarifying the limitations of this study and the empirical elements left open for future investigation in this article.

The first limitation of this study is the data analysis approach, considering only direct effects and not a path or moderation model (Sung et al., 2016). The important implication of this limitation is for the *collaboration and leadership* domain, which loses its influence in the final model when all the domains are considered. The fact that these are antecedents or contextual factors opens the way for more in-depth investigations through structural equation modeling in order to identify the paths through which these variables might affect the outcome of interest. Such an approach could identify not only the factors that influence teachers’ use of ICT, but also the path through which schools and policymakers can enhance this use (Liu et al., 2020).

In relation to this, a second limitation of this study is the data analysis exclusively addressing the teacher level. This approach does not allow us to account for the schools’ and leaders’ perspectives. The leaders’ perspective would be useful in particular to corroborate the teachers’ perceptions of *collaboration and leadership* in the school, but also the availability of ICT materials and ICT priorities. Having a school level in the model would allow for testing the level of teacher agreement within schools in terms of collaboration and leadership, in addition to individual perceptions (Costa et al., 2021; Howard et al., 2020). Moreover, having a school level would allow us to test which schools are more “in balance” in terms of support, cooperation, and vision, and to examine if these schools show better results in terms of ICT use in teaching. School profile analysis could be another approach to identify a typology of school types (Drossel et al., 2020) characterized by different levels of the basic elements.

This brings us to a third limitation, which is the choice of data analysis approach. Taking into consideration the contextual domain of *collaboration and leadership* of the *Four in Balance* model, as well as the nested structure of the data, multilevel data analysis may appear the most appropriate approach. However, the intra-class correlation coefficient of this data is only 0.05. This small value of ICC indicates that the amount of variance in the use of ICT between schools is relatively small, whereas the variance between teachers within schools is much larger. Therefore, our data analysis considered only the teacher level, accounting statistically for the nested structure of the data by using the IEA IDB Analyzer. As a robustness check of the results, we also ran the same model using multilevel data analysis, the results of which are presented in Appendix 2 of this paper. No change in the relationships of interest here appeared when using multilevel data analysis, but future research could consider this approach if the focus is explicitly on the role of schools and leadership.

A fourth limitation is related to the high level of teacher nonresponse in Luxembourg, as well as in other countries in the ICILS 2018 study (Fraillon et al., 2020b). For our purposes, teacher and school weights were re-estimated using observed demographic characteristics to work with a more precise population estimate for the teacher population. The risk of nonresponse bias determined by the unobserved variables related to the use of ICT could still be present. We acknowledge that we still do not know whether teachers’ non-response is related to the variables of interest. For example, we do not know if teachers who do not share a positive vision of ICT refuse to participate more than others or alternatively have a higher proportion of participating because they want to make their voice heard. Such limitations remain present in the data, although the alternative weighting approach helps in some ways. However, we see that Luxembourg teachers show a similarly low participation rate in other international studies (e.g., International Civic and Citizenship Study 2009), being the only two large-scale studies that required teacher participation in Luxembourg. In addition, the very small number of secondary schools in Luxembourg determines that all national and international studies ask for the participation of all schools in all studies, resulting in a very high demand for participation. It is common for schools and different participants within schools to decide not to participate in one or another study, most probably regardless of the topic. The later remark is also supported by the fact that we do not see any type of pattern in the school nonresponse in the ICILS 2018 by type of secondary school or educational track.

A fifth limitation is related to the data and the results being based on self-reports, which mostly indicate teachers’ attitudes and perceptions of their knowledge and skills. Hämäläinen et. al. (2021) found that teachers’ self-reported levels of skills are conditioned by what they consider to be adequate for their tasks. The authors used both actual measurements of teachers’ digital skills from PIAAC data and self-perceived levels of digital skills from TALIS data, and found that some teachers reported an adequate level of skills but exhibited low skills. This suggests that under specific expectations, a lower level of skill is considered adequate. In the current study, we did not work with the teachers’ levels of skills and competence, but instead aimed to identify what could support teachers’ use of ICT for teaching practice, with a focus on teachers’ attitudes, self-perceived efficacy, and use. This limitation concerning the measurements suggests caution should be exercised with regard to the expectation that a higher perceived level of ICT use in teaching will be positively associated with student learning and remedial teaching, considering that teachers with weak measured skills also perceived to be able to support student learning through digital technology (Hämäläinen et al., 2021).

Considering that the ICILS 2018 data allows for a very good coverage of the *Four in Balance* model, it would be relevant to apply such a model to other countries that participated in the study, especially those where teachers report a high use of ICT in teaching practice and that might have taken an “education-driven” approach (e.g., Denmark, Finland). Denmark and Finland would be very interesting cases to study further, considering that both countries have also ensured the adequate investment in ICT infrastructure. In Denmark, ICT resources and learning platforms have been widespread for a long time (Strietholt et al., 2021). In Finland, ICT equipment and learning materials for teaching have also been made available since 2010, through the Basic Education Act (Strietholt et al., 2021). Both countries nevertheless, set a great deal of importance on ICT-related professional development opportunities for teachers, as has also been the case during the COVID-19 health crisis. Furthermore, it would also be important to identify which domain or domains prove significant in predicting teachers’ pedagogical use of ICT, and especially if the impact of domains differs per innovation approach or other contextual factors. Testing this model across all countries participating in ICILS 2018 and later in ICILS 2023 would allow for a more in-depth understanding of how teachers’ use of ICT could be supported by schools and policymakers, and furthermore, how such approaches to ICT innovation would relate with student performance.

### Supplementary Information

**Additional file 1.** The alternative weighting approach.

## Data Availability

The ICILS 2018 data for Luxembourg is public under the IEA Data Repository. The additional final teacher weights generated for the alternative weighting approach can be made available on request.

## Appendix 1. List of scales and single items (Mikheeva & Meyers, 2020)

### Dependent variable

**Table:**

*Scale*: teacher use of ICT for teaching practices in class (T_ICTPRAC)
Items measured on a 4-point Likert scale ranging from “I never use ICT in this practice” to “I always use ICT in this practice”; built as an IRT WLE score with mean 50 and standard deviation 10, higher values indicating more frequent use
*How often do you use ICT in the following practices when teaching your reference class?*
The provision of remedial or enrichment support to individual students or small groups of students
The support of student-led whole-class discussions and presentations
The assessment of students’ learning through tests
The provision of feedback to students on their work
The reinforcement of learning of skills through repetition of examples
The support of collaboration among students
The mediation of communication between students and experts or external mentors
The support of inquiry learning

### Independent variables

#### ICT infrastructure

**Table:**

Availability of computer resources at school (T_RESRC)
Items measured on a 4-point Likert scale ranging from “Strongly disagree” to “Strongly agree”; built as two IRT WLE scores with mean 50 and standard deviation 10, higher values indicating stronger agreement
*To what extent do you agree or disagree with the following statements about using ICT in teaching at your school?*
My school has sufficient ICT equipment (e.g., computers)
The computer equipment in our school is up to date
My school has access to sufficient digital learning resources (e.g., learning software or [apps])
My school has good connectivity (e.g., fast speed and Stable) to the Internet
There is enough time to prepare lessons that incorporate ICT
There is sufficient opportunity for me to develop expertise in ICT
There is sufficient technical support to maintain ICT resources

#### Digital learning materials

**Table:**

*Scale:* use of digital learning tools (T_USETOOL)
Items measured on a 4-point Likert scale ranging from “Never” to “In every or almost every lesson”; built as IRT WLE scores with mean 50 and standard deviation 10, higher values indicating more frequent use
*How often did you use the following tools in your teaching of the reference class this school year?*
Practice programs or apps where you ask students questions (e.g., [Quizlet, Kahoot], [mathfessor])
Digital learning games
Concept mapping software (e.g., [Inspiration®], [Webspiration®])
Simulations and modeling software (e.g., [NetLogo])
A learning management system (e.g., [Edmodo], [Blackboard])
Collaborative software (e.g., [Google Docs®], [Onenote], [Padlet])
Interactive digital learning resources (e.g., learning objects)
Graphics or drawing software
e-portfolios (e.g., [VoiceThread])
Social media (e.g., [Facebook, Twitter])

**Table:**

*Scale*: use of general utility software (T_USEUTIL)
Items measured on a 4-point Likert scale ranging from “Never” to “In every or almost every lesson”; built as IRT WLE scores with mean 50 and standard deviation 10, higher values indicating more frequent use
*How often did you use the following tools in your teaching of the reference class this school year?*
Word-processing software (e.g., [Microsoft Word®])
Presentation software (e.g., [Microsoft PowerPoint®])
Computer-based information resources (e.g., topic-related websites, wikis, encyclopedia)
Digital content linked with textbooks

#### Expertise

**Table:**

*Single item*: experience with ICT use during lessons
Item measured on a 4-point Likert scale ranging from “Never” to “More than 5 years.”
*Approximately how long have you been using ICT for teaching purposes?*
*During lessons:*

**Table:**

*Single item*: initial teacher education on ICT
Two items measured with “Yes” or “No.”
*Did your [initial teacher education] include the following elements?*
(a) *Learning how to use ICT*
(b) *Learning how to use ICT in teaching*
For this study, the two items were collapsed into one:
*Did your [initial teacher education] include the following elements?*
Measured with “None,” “Either (a) or (b),”, and “Both (a) and (b)”

**Table:**

*Scale*: teacher ICT self-efficacy (T_ICTEFF)
Items measured on a 3-point Likert scale ranging from “I do not think I could do this” to “I know how to do this”; built as IRT WLE scores with mean 50 and standard deviation 10, higher values indicating higher level of self-efficacy
*How well can you do these tasks using ICT?*
Find useful teaching resources on the Internet
Contribute to a discussion forum/user group on the Internet (e.g., a wiki or blog)
Produce presentations (e.g., [PowerPoint® or a similar program]), with simple animation functions
Use the Internet for online purchases and payments
Prepare lessons that involves the use of ICT by students
Using a spreadsheet program (e.g., [Microsoft Excel®]) for keeping records or analyzing data
Assess student learning IT2G07G
Collaborate with others using shared resources such as [Google Docs®], [Padlet]
Use a learning management system (e.g., [Moodle], [Blackboard], [Edmodo])

#### Vision

**Table:**

*Scale*: positive views on using ICT in teaching and learning (T_VWPOS)
About the use of ICT for knowledge transmission and knowledge construction
Items measured on a 4-point Likert scale ranging from “Strongly disagree” to “Strongly agree”; built as IRT WLE scores with mean 50 and standard deviation 10, higher values indicating stronger agreement
*To what extent do you agree or disagree with the following practices and principles in relation to the use of ICT in teaching and learning?*
*Using ICT at school:*
Helps students develop greater interest in learning
Helps students to work at a level appropriate to their learning needs
Helps students develop problem-solving skills
Enables students to collaborate more effectively
Helps students develop skills in planning and self-regulation of their work
Improves the academic performance of students
Enables students to access better sources of information

#### Collaboration and leadership

**Table:**

*Scale*: collaboration between teachers in using ICT (T_COLICT)
Items measured on a 4-point Likert scale ranging from “Strongly disagree” to “Strongly agree”; built as IRT WLE scores with mean 50 and standard deviation 10, higher values indicating stronger agreement
*To what extent do you agree or disagree with the following statements about your use of ICT in teaching and learning at your school?*
I work together with other teachers on improving the use of ICT in classroom teaching
I collaborate with colleagues to develop ICT-based lessons
I observe how other teachers use ICT in teaching
I discuss with other teachers how to use ICT in teaching topics
I share ICT-based resources with other teachers in my school

**Table:**

*Scale*: teacher participation in collaborative reciprocal professional learning development related to ICT (T_PROFREC)
Items measured on a 3-point Likert scale ranging from “Not at all” to “More than once”; built as IRT WLE scores with mean 50 and standard deviation 10, higher values indicating more frequent participation
*How often have you participated in any of the following professional learning activities in the past two years?*
Observations of other teachers using ICT in teaching
An ICT-mediated discussion or forum on teaching and learning
The sharing of digital teaching and learning resources with others through a collaborative workspace
Use of a collaborative workspace to jointly evaluate student work

**Table:**

*Single item*: ICT as a school priority
Item measured on a 4-point Likert scale ranging from “Strongly disagree” to “Strongly agree.”
*To what extent do you agree or disagree with the following statements about using ICT in teaching at your school?*
ICT is considered a priority for use in teaching

## Appendix 2. Robustness check of the results

### Multilevel data analysis approach

Due to the two-level character of the data, with teachers nested within schools (Snijders & Bosker, 2012), we also used hierarchical linear modeling (HLM) to investigate the Four in Balance proxy model.

The HLM statistical package was used and the ICILS 2018 specific teacher and school weights were composed for the multilevel approach (the product of the alternative weighting approach teacher/school weight factor and the alternative weighting approach teacher/school weight adjustment).

As indicated before, the empty model shows that most of the variance occurs at the teacher level, with only 5 percent of the variance to be explained being at the school level. When running all models following the same approach as previously presented (see Table 6), we find the same results in terms of the size and direction of the significant relationships, and their relative contribution to the explained variance of all models. No additional variables were tested (only the *Four in Balance* proxy model) for cross-validation, as the introduction of school background characteristics determined the reduction of the school sample to 20, due to nonresponse or missing data at the school level.

## Footnotes

1 In Luxembourg, two explicit strata were defined prior to school sampling: public schools or following the national curriculum and private schools.
