# Supplementary material for: Implementing ICT in classroom practice: what else matters besides the ICT infrastructure?
Source: Large Scale Assess Educ. 2023 Jan 13;11(1):1. doi: 10.1186/s40536-022-00144-6 (PMC9837759; doi:10.1186/s40536-022-00144-6)
Supplement: Supplementary file 1 — Additional file 1. The alternative weighting approach. [file 40536_2022_144_MOESM1_ESM.docx]

**Additional file 1**

**Re-weighting of the ICILS 2018 Luxembourg teacher data**

***Preparation of the teacher datasets for re-weighting***

To summarize the next results, the following characteristics of teachers show differences in answers: the teacher’s subject domain (measured as language and arts, human sciences, mathematics and sciences, other, and missing), the teacher’s age group (original teacher age groups with mid age in parenthesis: (27) less than 29 years old, (35) 30 to 39 years old, (45) 40 to 49 years old, (55) 50 to 59 years old, (63) 60 years old or above, and missing (99)), and the school type (public schools following the national curriculum, private schools following the national curriculum, and private schools not following the national curriculum). These characteristics were used to define the alternative nonresponse cells. The subject domain and age group were already available for each teacher in participating schools, but missing for three schools that initially declined to participate for different reasons. The school type was indicated at national level as a characteristic of each participating school.^[[1]](#footnote-1)^

In a first step, the alternative adjustment cells need to be defined, so that the weights for non-participating teachers are distributed between participating teachers within the same group. Based on analyzing the characteristics of the sample of teachers who answered the questionnaire and comparing this with the population of teachers, we see an over or under-representation of specific characteristics in the sample of teachers who answered. More specifically, some subject domains are over or under-represented in the sample (23 percent mathematics and science teachers in the sample compared with 19 percent in the population, and 13 percent of human sciences teachers in the sample compared with 15 percent in the population), age groups (notably older teachers, with 19 percent in the sample compared with 23 percent in the population), and school type (fewer schools not following the national curriculum in the sample of teachers who answered compared with the teacher population).

Using all the combinations of these characteristics would have led to about 65 adjustment cells with at least one teacher, only 12 of these containing more than 20 teachers, and the remaining cells without any teachers. Consequently, the first step was to define which of the smaller cells could be collapsed.

- The school type was reduced to two levels, as teachers in schools (either public or private) following the national curriculum, and teachers in (private) schools not following the national curriculum showed different answer behaviors.
- The subject domain “other” was combined with “missing,” resulting in four levels. These cells contain a relatively small number of teachers and it was expected that teachers within these groups would answer differently.
- With regard to the age groups, the age groups (55) 50 to 59 years old and (63) 60 years old and above were collapsed as the numbers of teachers in both age groups were relatively small, and we assume that teachers in both groups gave similar answers. Further age groups were also collapsed when considering the school type level. For schools following the national curriculum, the age groups (35) 30 to 39 years old and (99) “missing” were collapsed, as the former contained most teachers and the number of teachers in the missing age group was small. For schools not following the national curriculum, age groups (27) under 29 years old and (35) 30 to 39 years old were collapsed, as the number of teachers was relatively small in these cells and their answers are expected to be similar.

This resulted in 28 adjustment cells (see Table S1 here). Each teacher group is defined by the school type (second column), the (combined) subject domain(s) (third column), and the (combined) teacher age group(s) (forth column). For each teacher group, the number of teachers is given in the fifth column. The nonresponse adjustment cells for the schools not following the national curriculum (rows 17–28) are fairly small, so condensing all the age groups within subject domains 2 to 4 would also be reasonable. To have a similar approach as that for the schools following the national curriculum, we retained these very small nonresponse adjustment cells.

Table S1: Examples of alternative nonresponse adjustment cells

| **Nonresponse adjustment cell No.** | **School type** | **Subject Domain** | **Age group** | **Number of teachers for each group** |
| --- | --- | --- | --- | --- |
| 1 | Schools following national curriculum | 1 | 27 | 33 |
| 2 | Schools following national curriculum | 1 | 35 and 99 | 142 |
| 3 | Schools following national curriculum | 1 | 45 | 79 |
| 4 | Schools following national curriculum | 1 | 55 and 63 | 55 |
| 5 | Schools following national curriculum | 2 | 27 | 10 |
| 6 | Schools following national curriculum | 2 | 35 and 99 | 61 |
| 7 | Schools following national curriculum | 2 | 45 | 30 |
| 8 | Schools following national curriculum | 2 | 55 and 63 | 23 |
| 9 | Schools following national curriculum | 3 | 27 | 16 |
| 10 | Schools following national curriculum | 3 | 35 and 99 | 62 |
| 11 | Schools following national curriculum | 3 | 45 | 35 |
| 12 | Schools following national curriculum | 3 | 55 and 63 | 28 |
| 13 | Schools following national curriculum | 4 | 27 | 11 |
| 14 | Schools following national curriculum | 4 | 35 and 99 | 71 |
| 15 | Schools following national curriculum | 4 and 9 | 45 | 60 |
| 16 | Schools following national curriculum | 4 and 9 | 55 and 63 | 60 |
| 17 | Schools not following national curriculum | 1 | 27 and 35 | 30 |
| 18 | Schools not following national curriculum | 1 | 45 | 19 |
| 19 | Schools not following national curriculum | 1 | 55 and 63 | 21 |
| 20 | Schools not following national curriculum | 2 | 27 and 35 | 6 |
| 21 | Schools not following national curriculum | 2 | 45 | 5 |
| 22 | Schools not following national curriculum | 2 | 55 and 63 | 6 |
| 23 | Schools not following national curriculum | 3 | 27 and 35 | 12 |
| 24 | Schools not following national curriculum | 3 | 45 | 10 |
| 25 | Schools not following national curriculum | 3 | 55 and 63 | 11 |
| 26 | Schools not following national curriculum | 4 | 27 and 35 | 10 |
| 27 | Schools not following national curriculum | 4 | 45 | 12 |
| 28 | Schools not following national curriculum | 4 | 55 and 63 | 8 |

## ***Weighting factors***

Using the teacher groups as described above requires the most weighting factors needing to be updated.

## *School base weight – WGTFAC1T*

The school base weight is the reciprocal of the sampling probability. For Luxembourg, each school $i$ was asked to participate. Thus, the selection probability and therefore the school base weight equals 1 for all schools, regardless of the stratum (see equation A.1):

${WGTFAC1T}_{i}=1$ (A.1)

### *School nonresponse adjustment – WGTADJ1T*

In large-scale assessments, nonresponding schools are accounted for by the school nonresponse adjustment, which enlarges the weights for the participating schools and is calculated per explicit stratum. For the alternative weighting approach for Luxembourg, the distinction between schools following the national curriculum and schools not following the national curriculum was used as the explicit stratum. This was possible, because there is a census of schools in Luxembourg.

The school nonresponse adjustment $WGTADJ1T$ is calculated for each participating school $i$ in explicit stratum $h$ by dividing the number of sampled, eligible^[[2]](#footnote-2)^ schools $n_{h}^{s,e}$ by the number of participating schools $n_{h}^{p}$ within each explicit stratum $h$ (see equation A.2):

${WGTADJ1T}_{hi}=n_{h}^{s,e}/n_{h}^{p}$ (A.2)

Two schools that were not following the national curriculum and one school that followed the national curriculum did not participate from the beginning. This results in (equation A.3 and A.4):

${WGTADJ1T}_{school i following the national curriculum}=33/32= 1.03125$ (A.3)

and

${WGTADJ1T}_{school i not following the national curriculum}=8/6=1.3333$ (A.4)

In one additional school following the national curriculum, no teacher participated, although all the teacher sampling information was provided (based on the ICILS 2018 sampling procedure). These nonparticipating teachers were accounted for but by WGTADJ2T instead of WGTADJ1T.

### *Teacher base weight – WGTFAC2S*

The teacher base weight is the reciprocal of the sampling probability. For Luxembourg, 25 teachers in each school $i$ in stratum $h$ were asked to participate, thus the teacher base weight for each teacher $l$ equals:

${WGTFAC2T}_{hil}=T_{hi}/25$ (A.5)

where $T_{hi}$ is the total number of eligible teachers in school $i$ in stratum$h$.

### *Teacher multiplicity factor – WGTFAC3T*

Some teachers in ICILS 2018 were teaching at the target grade in more than one school (based on information from the teacher questionnaire), and therefore had a larger selection probability than their equivalents teaching in only one school. To account for this, a “teacher multiplicity factor” was calculated as the inverse of the number of schools in which the teacher was working:

${WGTFAC3T}_{hil}=1/f_{hil}$ (A.6)

Here, $f_{hil}$ is the number of schools where teacher $l$ in school $i$ in stratum$h$ was teaching.

The teacher multiplicity factor is not affected by the alternative weighting approach. However, it needed to be updated for teachers accounted for as “participating” in this alternative weighting approach, but accounted for as “not participating” in the ICILS 2018 international database.

### *Teacher nonresponse adjustment – WGTADJ2T*

The teacher nonresponse adjustment $WGTADJ2T$ accounts for non-participating teachers within the defined adjustment cells. For the international ICILS 2018 database, the nonresponse cell for non-participating teachers is the school, and for the alternative weighting approach, it is the new adjustment cells defined. Therefore, this weight factor was calculated for each participating teacher $l$ within age group $a$, and with subject domain $s$ in the new defined stratum $h$:

${WGTADJ2T}_{hsal}=t_{hsa}^{s,e}/t_{hsa}^{p}$ (A.7)

where $t_{hsa}^{s,e}$ is the number of sampled teachers, and $t_{hsa}^{p}$ is the number of participating teachers in age group $a$ and subject domain $s$ in stratum $h$. Teachers who left a school after they had been sampled, but prior to the data collection, were regarded as out of scope. Thus, their weights were not adjusted in these instances.

It should be noted that for the ICILS 2018 international database, within a nonresponse cell it is required that at least 50 percent of the units participate. Therefore, in a school where less than 50 percent of the sampled teachers participated, all teachers were deemed as non-participating and were not part of the international database. This applies to 10 schools in the international database and 354 teachers who did not answer, but also to 79 teachers who answered. This resulted into a total teacher sample of 573 teachers who answered the questionnaire.

Following the rules for non-participation—as applied in international large-scale assessments—at least 50 percent of the teachers per teacher group need to participate. However, this would lead to rejecting the data for two smaller non-response cells (nr. 8 and nr. 16, i.e. with age groups 55/63). In order not to miss out the information for these age groups, we retained them, but should comment that these groups have a teacher nonresponse adjustment factor between 2 and 3. Thus, the results for these teacher groups might be biased.

***Final teacher weight – TOTWGTT***

The final weight, TOTWGTT, for teacher $l$ within age group $a$, and with subject domain $s$ in school $i$ in the new defined stratum $h$ is then

$\mathrm{TOTWGTT}_{hisal} {= WGTADJ1T}_{hi}*{{WGTFAC2T}_{hil}*WGTADJ2S}_{hsal}*{WGTFAC3S}_{hil}$ (A.8)

Please note that ${WGTFAC1T}_{i}$ is left out of this formula, as it equals 1.

***Calculation of the jackknife variables for variance estimation***

Lastly, the Jackknife variables needed to be updated. The values of the Jackknife zones and Jackknife replicates were in general kept, as no changes were necessary. However, for those teachers considered as participating in the present alternative weighting approach, respective Jackknife zones and Jackknife replicates were assigned following the same procedure as in the international data.

To be able to use the IDB Analyzer for further analysis, a new dataset was created including the new calculated teacher weight, jackknife zone, jackknife replicate, and all replicate weights. Comparing the final teacher weights of the ICILS 2018 international database with the final teacher weights of the alternative weighting approach shows that these are similar (r^2^ = .70). However, the sum of the final teacher weights is slightly higher than that of the international database. According to figures given prior to school sampling, this new sum is a more precise population estimate for the teacher population.

1. For the ICILS 2018 international database, the school administration (public vs. private) was used as the explicit stratum. The school type, used here for calculating the alternative weights, was not used to calculate the initial IEA international data weights. [↑](#footnote-ref-1)
2. Note that for Luxembourg, all the sampled schools were eligible. [↑](#footnote-ref-2)
